# Effects of soy foods on ovarian function in premenopausal women

**DOI:** 10.1054/bjoc.1999.1218

**Published:** 2000-06

**Authors:** A H Wu, F Z Stanczyk, S Hendrich, P A Murphy, C Zhang, P Wan, M C Pike

**Affiliations:** Departments of Preventive Medicine, Obstetrics and Gynecology, University of Southern California, School of Medicine, Los Angeles, California, USA; Food Science and Human Nutrition, Iowa State University, Ames, IA, USA

**Keywords:** soy foods, ovarian function, premenopausal women, intervention study

## Abstract

It has been proposed that the high intake of soy foods among Asians may partly explain their lower rates of breast cancer, perhaps by lowering endogenous oestrogen levels, although this has been inadequately studied. Twenty healthy cycling premenopausal women (ten Asians and ten non-Asians) participated in a 7-month soy intervention study which was designed to investigate the effect of supplementation on ovarian function. Asian soy foods (tofu, soymilk, green soybean peas) in the amount of approximately 32 mg of isoflavones per day were added to the women's diets for three menstrual cycles. The women's baseline (two cycles) serum hormone levels were compared to levels during soy intervention (three cycles) and levels after intervention (two cycles). During the entire study period, subjects provided almost daily overnight urine samples and blood specimens during specified days of their menstrual cycles. The day of urinary luteinizing hormone (LH) peak was used as a marker for the day of ovulation. Knowledge of day of ovulation allowed comparison of hormone measurements at baseline to those obtained during intervention and recovery cycles with standardization of day of cycle. Soy intervention was associated with a statistically significant reduction in serum luteal oestradiol level (–9.3%, *P*< 0.05), but there were no significant changes in follicular phase oestradiol, follicular or luteal phase progesterone, sex hormone-binding globulin or menstrual cycle length. This significant reduction in luteal phase oestradiol was, however, observed only among Asian (–17.4%) but not among non-Asian (–1.2%) participants; urinary excretion of isoflavones was higher among Asians than non-Asians (29.2 vs 17.1 μmol day^−1^, *P* = 0.16) during the intervention period. Thus, supplementation using traditional soy foods reduced serum oestradiol levels among Asian participants in this study. Differences in the type of soy products (i.e. traditional soy foods versus soy protein products), amount of isoflavones, and race/ethnicity of participants may have contributed to the divergent results. Larger soy intervention studies designed specifically to include participants of different race/ethnicities and using both traditional soy foods and soy protein products providing comparable doses of isoflavones are needed to definitively determine the effect of soy on ovarian function. © 2000 Cancer Research Campaign


British Journal of Cancer (2000) 82(**11**), 1879–1886
© 2000 Cancer Research Campaign
DOI: 10.1054/ bjoc.1999.1218, available online at http://www.idealibrary.com on IDEAL®

### Effects of soy foods on ovarian function in premenopausal women

**AH Wu[1], FZ Stanczyk[2], S Hendrich[3], PA Murphy[3], C Zhang[2], P Wan[1] and MC Pike[1]**

Departments of [1]Preventive Medicine and [2]Obstetrics and Gynecology, University of Southern California, School of Medicine, Los Angeles, California, USA; [3]Food Science and Human Nutrition, Iowa State University, Ames, IA, USA.

**Summary** It has been proposed that the high intake of soy foods among Asians may partly explain their lower rates of breast cancer, perhaps by lowering endogenous oestrogen levels, although this has been inadequately studied. Twenty healthy cycling premenopausal women (ten Asians and ten non-Asians) participated in a 7-month soy intervention study which was designed to investigate the effect of supplementation on ovarian function. Asian soy foods (tofu, soymilk, green soybean peas) in the amount of approximately 32 mg of isoflavones per day were added to the women's diets for three menstrual cycles. The women's baseline (two cycles) serum hormone levels were compared to levels during soy intervention (three cycles) and levels after intervention (two cycles). During the entire study period, subjects provided almost daily overnight urine samples and blood specimens during specified days of their menstrual cycles. The day of urinary luteinizing hormone (LH) peak was used as a marker for the day of ovulation. Knowledge of day of ovulation allowed comparison of hormone measurements at baseline to those obtained during intervention and recovery cycles with standardization of day of cycle. Soy intervention was associated with a statistically significant reduction in serum luteal oestradiol level (–9.3%, $P < 0.05$), but there were no significant changes in follicular phase oestradiol, follicular or luteal phase progesterone, sex hormone-binding globulin or menstrual cycle length. This significant reduction in luteal phase oestradiol was, however, observed only among Asian (–17.4%) but not among non-Asian (–1.2%) participants; urinary excretion of isoflavones was higher among Asians than non-Asians (29.2 vs 17.1 μmol day$^{-1}$, $P = 0.16$) during the intervention period. Thus, supplementation using traditional soy foods reduced serum oestradiol levels among Asian participants in this study. Differences in the type of soy products (i.e. traditional soy foods versus soy protein products), amount of isoflavones, and race/ethnicity of participants may have contributed to the divergent results. Larger soy intervention studies designed specifically to include participants of different race/ethnicities and using both traditional soy foods and soy protein products providing comparable doses of isoflavones are needed to definitively determine the effect of soy on ovarian function. © 2000 Cancer Research Campaign

**Keywords**: soy foods; ovarian function; premenopausal women; intervention study

Historically, breast cancer rates in Asia are one-sixth of the rates of Whites in the USA. Corresponding to the lower breast cancer rates in Asia, endogenous oestradiol levels are also lower among native Asians compared to White women in the USA or UK (Wu and Pike, 1995). Reasons for the lower endogenous oestradiol levels and lower breast cancer rates in Asians are not known. One hypothesis is that the traditionally high intake of soy in Asia, the main source of isoflavones (one of two main classes of phyto-oestrogens in the human diet), may explain in part the lower breast cancer rates and oestrogen levels (Adlercreutz, 1990; Messina and Barnes, 1991). The mechanisms by which soy influences risk of breast cancer are not known. A predominant hypothesis focuses on the potential effects of soy on hormone production and metabolism. There is currently only circumstantial evidence in support of this hypothesis. A small cross-sectional study of premenopausal women in Japan reported an inverse association between intake of soy foods and serum oestradiol levels (Nagata et al, 1997). Two (Lu et al, 1996; Nagata et al, 1998) of five (Cassidy et al, 1994; Lu et al, 1996; Petrakis et al, 1996; Nagata et al, 1998; Duncan et al, 1999) soy intervention studies in premenopausal women found a substantial reduction in serum oestradiol levels in association with soy supplementation. Both 'positive' studies (Lu et al, 1996; Nagata et al, 1998) used soy foods (instead of soy protein or isolates) and higher amounts of isoflavones than the other studies (Duncan et al (1999) used two doses; the higher dose was comparable to that used in Nagata et al (1998)). We designed a study to further investigate the effects of soy on ovarian function in premenopausal women using traditional Asian soy foods, as the protective effect observed in epidemiologic studies is based on populations consuming soy foods (not soy protein or isolates). As part of the study design, we also collected daily morning urine specimens during the entire study in order that the dates of ovulation (based on peak levels of urinary luteinizing hormone (LH)) could be accurately estimated. Knowledge of the day of ovulation allowed us to evaluate hormonal responses to soy supplementation after standardization for the day of blood specimen collection.

*Received 11 October 1999*
*Revised 29 November 1999*
*Accepted 14 December 1999*

*Correspondence to*: AH Wu, USC/Norris Comprehensive Cancer Center, 1441 Eastlake Avenue, MS#44, PO Box 33800, Los Angeles, CA 90089–9011, USA

### SUBJECTS AND METHODS

### Study subjects and data collection

Study participants were employees at the Health Sciences Campus at the University of Southern California. We advertised the study and held several soy food-tasting luncheons to describe the objectives of the study and requirements for participation. Interested subjects completed a brief questionnaire that included information on demographic and selected lifestyle characteristics. Exclusion criteria included current or recent (within the last 12 months) pregnancy or lactation, irregular menstrual cycles, current use of oral or other hormonal contraceptives or hormones, history of chronic illness (e.g. diabetes) or cancer, current smoker and following a special diet. Twenty women completed the study of at least seven menstrual cycles in a free-living environment.

The study protocol was approved by the University of Southern California Institutional Review Board Human Subjects Committee. The study consisted of a baseline period of two menstrual cycles, a soy intervention period of three cycles and a ‘recovery’ period of at least two cycles (eight of the 20 women completed an 8th month, i.e. had three recovery cycles). Throughout the duration of the study, participants were asked to consume their usual diets with added instructions to avoid intake of soy products during the baseline and recovery periods. During the soy intervention period, their usual diets were supplemented with three traditional Asian soy foods (tofu, soymilk and frozen soybeans peas (edamame)) purchased by the investigators. Subjects were instructed to consume 161 g of Mori-Nu Silken Extra Firm tofu or 322 g of Eden soymilk or 75 g of Kimbo frozen soybean peas per day; these amounts of the three foods contained equivalent amounts of isoflavones. On the basis of published soy food isoflavone levels (Coward et al, 1993), subjects were permitted to consume any combination of the three foods that provided an equivalent amount of isoflavone. The soy diet commenced on day 1 of the third menstrual cycle and ended on day 1 of the sixth menstrual cycle.

At study entry, subjects completed a questionnaire that asked about menstrual and reproductive history. During the study, subjects completed a daily log which itemized consumption of alcoholic beverages, duration of physical exercise and consumption of any soy products during the months when they were supposed to abstain from these foods. In addition, a daily ‘soy’ log was used to record the specific soy food and amount consumed during the soy intervention cycles. Four times during each menstrual cycle (typically one recall per week), a 24-h diet recall was completed. Average intakes of energy, total and components of fat, protein, carbohydrate, cholesterol, dietary fibre and other micronutrients were calculated for each menstrual cycle (food records were analysed at the Nutrition Service Core at the University of Hawaii).

Overnight daily voids were collected from participants during the study. Urine specimens were collected in plastic containers containing 1 g of ascorbic acid. Subjects were instructed to void prior to sleep and collect all subsequent voids including the first morning one. The period of urine collection was at least 4 h. A 100-ml aliquot of each urine specimen was taken for the study and the remainder was discarded. The 100-ml aliquot was subdivided into 6 portions of 15–20 ml and stored at –20°C. Creatinine (CR) was measured in all urine samples to standardize LH and isoflavone values. Blood specimens were obtained on two occasions (days 10–12 and days 20–22) of each menstrual cycle, allowed to clot and the serum was stored at –70°C. In the event that menstrual cycles exceeded 28 days, blood specimens were also collected on days 29, 36 and 43 (if applicable). Once per month at the time of blood collection, body weight and hip and waist measurements were obtained.

### Analysis of isoflavone levels in foods and urines

The same brand of soy foods was purchased and used during the entire study. The total isoflavone levels (all 12 isomers of daidzein, genistein, glycitein) in the soy foods (Mori-Nu Silken Extra Firm tofu, Kimbo frozen soybean peas, and Eden soymilk) were determined by one of us (PM) nine times during the study to assess variability in the amount of isoflavones in these foods. Four random urine samples (about one sample per week) per cycle were selected and tested for urinary isoflavone levels to determine compliance during the soy intervention period and avoidance of these foods during baseline and recovery (urinary isoflavone analysis was conducted by Drs Patricia Murphy and Suzanne Hendrich, Iowa State University). To determine both food and urinary isoflavone levels, we used a high-performance liquid chromatography (HPLC) quantitation method, developed by these investigators which hydrolyses isoflavone metabolites and extracts and quantifies isoflavone aglycones (Wang and Murphy, 1994; Xu et al, 1994; Murphy et al, 1997).

### Measurement of serum and urinary hormones

Hormone analyses were conducted by Dr Frank Z Stanczyk at the University of Southern California. Total oestradiol and progesterone were measured in serum by validated specific radioimmunoassay (RIA) methods following extraction (Scott et al, 1978; Stanczyk et al, 1988). Urinary LH was measured by an immunoradiometric assay using the LH MAIA clone kit (Biodata Diagnostics, Rome, Italy). SHBG was quantified by RIA utilizing a commercial kit obtained from Diagnostics Systems Laboratories (Webster, TX, USA). Because the objective of this study was to test for differences in hormone profiles within individuals, the urinary and serum hormone measurements for each participant was performed in the same assay to reduce inter-assay variability. Urinary creatinine (CR) levels were measured colorimetrically. Cholesterol and triglyceride measurements were conducted by a commercial laboratory (Endocrine Sciences, Calabasas Hills, CA, USA).

### Statistical analysis

### *Daily urinary isoflavone excretion*

Individual urinary isoflavone measurements were transformed logarithmically to achieve approximate normal distributions. We calculated the daily urine isoflavone excretion as (isoflavone concentration/CR concentration) $\times$ 1.2 g, where 1.2 g is the assumed daily creatinine excretion (Krupps et al, 1982).

### *Standardization of hormone values*

Our approach to ‘adjustment’ for day of the cycle was to calculate ‘standardized’ hormone values as follows. We first identified the day when the urinary LH/CR level was a clear maximum and assumed that day of ovulation was 1 day after this peak. The follicular phase was defined as day 1 of the menstrual cycle through the day of the LH/CR peak, and the luteal phase was defined as the remainder of the cycle through the last day before the start of the next menses. Based on the ‘ovulation’ day (day 0) for a specific subject and menstrual cycle, each blood specimen was assigned a ‘true’ specimen collection day relative to ovulation (i.e. days –1, –2, –3, –4, etc. were assigned to specimens collected prior to the day of ovulation, and days 0, +1, +2, +3, +4, etc. for those collected at or after the day of ovulation).

© 2000 Cancer Research Campaign

**Table 1** Body mass index, serum lipid profile and dietary intake (mean ± s.d.)

| | Subjects | Baseline | Intervention | Recovery |
|---|---|---|---|---|
| Body size | All | 23.1 ± 3.3 | 23.6 ± 3.5 | 23.7 ± 3.5 |
| BMI (kg $m^{-2}$) | Asians | 22.7 ± 4.1 | 23.1 ± 4.3 | 23.1 ± 4.4 |
| | Non-Asians | 23.6 ± 2.4 | 24.2 ± 2.5 | 24.4 ± 2.5 |
| Serum levels (mg $dL^{-1}$ | | | | |
| Total cholesterol | All | 169.6 ± 30.0 | 169.1 ± 26.7 | 170.7 ± 26.6 |
| | Asians | 162.5 ± 30.7 | 161.9 ± 28.3 | 162.4 ± 26.4 |
| | Non-Asians | 176.7 ± 29.0 | 176.1 ± 24.3 | 179.0 ± 25.4 |
| HDL cholesterol | All | 59.8 ± 15.0 | 59.7 ± 13.3 | 60.5 ± 13.0 |
| | Asians | 52.8 ± 10.9 | 53.6 ± 9.2 | 53.2 ± 8.9 |
| | Non-Asians | 66.7 ± 15.8 | 65.6 ± 14.3 | 67.6 ± 12.6 |
| LDL cholesterol | All | 92.3 ± 39.1 | 91.4 ± 40.0 | 90.1 ± 36.9 |
| | Asians | 96.9 ± 50.8 | 92.1 ± 50.3 | 95.3 ± 45.1 |
| | Non-Asians | 87.6 ± 24.6 | 90.3 ± 29.6 | 84.7 ± 28.1 |
| Triglycerides | All | 86.3 ± 26.8 | 85.8 ± 23.9 | 87.7 ± 22.9 |
| | Asians | 88.5 ± 30.6 | 87.3 ± 28.8 | 84.9 ± 27.1 |
| | Non-Asians | 84.1 ± 23.8 | 84.1 ± 19.2 | 90.6 ± 18.8 |
| Daily diet | | | | |
| % calories from fat | All | 33.2 ± 6.5 | 33.0 ± 4.8 | 33.4 ± 6.5 |
| | Asians | 31.4 ± 6.4 | 32.7 ± 4.5 | 32.5 ± 4.8 |
| | Non-Asians | 35.0 ± 6.3 | 33.3 ± 5.3 | 34.4 ± 8.1 |
| % calories from | All | 15.9 ± 2.3 | 16.7 ± 2.4 | 16.0 ± 2.3 |
| protein | Asians | 16.6 ± 2.7 | 17.5 ± 3.0 | 15.8 ± 2.8 |
| | Non-Asians | 15.3 ± 1.8 | 15.9 ± 1.5 | 16.3 ± 1.8 |
| % calories from | All | 51.0 ± 7.8 | 50.5 ± 5.0 | 50.8 ± 6.3 |
| carbohydrates | Asians | 52.7 ± 7.5 | 50.1 ± 5.3 | 52.1 ± 6.1 |
| | Non-Asians | 49.3 ± 8.2 | 50.8 ± 4.8 | 49.3 ± 6.5 |
| Dietary cholesterol | All | 248.9 ± 130.1 | 236.3 ± 131.9 | 223.6 ± 121.1 |
| (mg) | Asians | 300.7 ± 132.4 | 281.2 ± 140.7 | 244.3 ± 123.3 |
| | Non-Asians | 197.0 ± 107.6 | 184.2 ± 100.3 | 193.2 ± 94.1 |
| Dietary fibre (g) | All | 16.2 ± 4.3 | 18.6 ± 6.6 | 14.5 ± 5.1 |
| | Asians | 17.4 ± 3.8 | 19.2 ± 6.8 | 14.6 ± 5.2 |
| | Non-Asians | 15.0 ± 4.5 | 17.8 ± 6.4 | 14.3 ± 5.1 |

We then constructed a standard curve for oestradiol (and separately for progesterone and SHBG) by calculating the geometric mean of oestradiol values for each specific day of the menstrual cycle (as defined above) utilizing all of the specimens collected. (Geometric mean values were calculated rather than mean values because of the well-known skewed distribution of oestradiol values.) ‘Adjusted’ oestradiol values were then calculated as the ratio of the measured oestradiol values to the values of this standard curve at the same day of the cycle. Adjusted hormone measurements were transformed logarithmically to achieve approximate normal distributions.

To evaluate whether serum hormone levels change in association with soy intervention, *t*-tests and analysis of variance (ANOVA) were conducted to compare the averaged log standardized ratios during baseline months to that obtained during intervention and recovery months. Results shown in the tables are obtained by taking the exponential of the average difference in log standardized hormone levels between the study periods of interest.

A total of seven menstrual cycles (three intervention and four recovery months) were excluded from data analysis because urine specimens were unavailable for more than 3 days between days 10 and 22 of the menstrual cycle, and the LH peak could not be determined with confidence. Results are shown for analyses that included a total of 147 menstrual cycles (40 baseline, 57 intervention and 50 recovery). There were 13 other menstrual cycles (three baseline, seven intervention and three recovery) in which luteal serum progesterone levels were below 3.0 ng $ml^{-1}$ (a presumptive marker of ovulation) (Israel et al, 1972) (range 0.35–2.76). Seven of these 13 specimens were collected 3 days after ovulation and three others were collected more than 10 days after ovulation. Thus, the low progesterone levels of most of these samples may be related to the collection of samples either too soon or too late after ovulation. We repeated all analyses after exclusion of these 13 menstrual cycles (referred to as the ‘clean’ analysis which included data on 134 menstrual cycles (37 baseline, 50 intervention and 47 recovery)).

### RESULTS

The mean age of the 20 study participants was 34.1 ± 7.4 years (range 21–44). An equal number of parous and nulliparous women (mean ages were 37.3 and 30.9 respectively) and an equal number of Asian and non-Asian (mean ages were 35.6 and 32.6 respectively) participants were included. (Asians included six Chinese, one Japanese, two Vietnamese and one Filipino; and non-Asians included six whites and four Latinas.) There were only very slight changes in body measurements during the study period in all subjects combined, or separately in Asians and non-Asians (Table 1, data for circumferences of waist and hip and weight not shown). Similarly, there were no significant changes in total serum cholesterol or any of the lipid fractions during the study period (Table 1). Intake of energy, fat, protein, carbohydrates (both

*© 2000 Cancer Research Campaign*

**Table 2** Mean daily urinary excretion of total isoflavones/creatinine [a](μmol day$^{-1}$)

| Subjects | Baseline* Mean ± s.e. | Intervention* Mean ± s.e. | Recovery Mean ± s.e. |
|---|---|---|---|
| All | 0.34 ± 1.17 | 20.28 ± 1.26[b] | 2.69 ± 0.56 |
| Asians | 0.31 ± 1.28 | 32.07 ± 1.27† | 3.87 ± 0.66† |
| Non-Asians | 0.36 ± 1.22 | 12.81 ± 1.42† | 1.52 ± 0.27† |

[a]Geometric means of total urinary isoflavone (daidzein, genistein, glycetein)/creatinine are shown. [b]Geometric means of daidzein, genistein, glycetein (divided by creatinine) were 11.34, 6.39 and 1.27 respectively for all subjects combined; the corresponding levels were 17.00, 11.65 and 1.66 in Asian participants, and 7.56, 3.50 and 0.97 in non-Asian participants. *$P < 0.05$ for difference in total urinary isoflavones/creatinine between baseline and intervention levels, for all subjects combined and separately in Asians and non-Asians. †$P < 0.05$ for difference in total urinary isoflavones/creatinine between Asians and non-Asians for the specific study period.

**Table 3** Serum estradiol, progesterone and sex hormone-binding globulin levels during intervention and recovery months relative to baseline levels

| Hormone | Subjects | Intervention/ Baseline (%) | *P*-value | Recovery/ Baseline (%) | *P*-value |
|---|---|---|---|---|---|
| Follicular oestradiol | All | 99.3 | 0.92 | 90.5 | 0.32 |
| | Asians | 92.6 | 0.40 | 89.4 | 0.39 |
| | Non-Asians | 106.0 | 0.54 | 91.4 | 0.58 |
| Luteal oestradiol | All | 90.7 | 0.05 | 93.2 | 0.43 |
| | Asians | 82.6 | 0.005 | 97.6 | 0.79 |
| | Non-Asians | 98.8 | 0.87 | 88.8 | 0.47 |
| Follicular progesterone | All | 99.4 | 0.96 | 106.4 | 0.55 |
| | Asians | 96.5 | 0.84 | 101.5 | 0.94 |
| | Non-Asians | 102.4 | 0.79 | 110.7 | 0.31 |
| Luteal progesterone | All | 86.1 | 0.43 | 71.9 | 0.20 |
| | Asians | 81.2 | 0.33 | 77.4 | 0.17 |
| | Non-Asians | 90.9 | 0.77 | 66.3 | 0.43 |
| Follicular SHBG | All | 98.5 | 0.80 | 98.1 | 0.78 |
| | Asians | 85.1 | 0.08 | 94.9 | 0.68 |
| | Non-Asians | 118.0 | 0.009 | 101.0 | 0.89 |
| Luteal SHBG | All | 98.0 | 0.75 | 94.3 | 0.38 |
| | Asians | 87.7 | 0.18 | 94.9 | 0.68 |
| | Non-Asians | 116.4 | 0.05 | 96.6 | 0.65 |

absolute intakes and as per cent of calories), fibre and various micronutrients, based on dietary recalls, also did not change significantly during the study period (Table 1).

On a per gram basis (wet weight), the total isoflavone (all 12 isomers of daidzein, genistein and glycitein) content found in our test foods was as follows: highest in tofu (0.247 mg g$^{-1}$), intermediate in soybean peas (0.197 mg g$^{-1}$) and lowest in soymilk (0.075 mg g$^{-1}$). On the basis of these results, the isoflavone content of 1 g of tofu is approximately equivalent to 1.2 g of soybeans or 3.3 g of soymilk. Using these isoflavone content values, we calculated that the mean daily isoflavone intake among study participants during the intervention months was 32.0 ± 10.5 mg (36.2 ± 12.0 mg in Asians, and 27.7 ± 7.0 mg in non-Asians, $P = 0.07$).

Each specific urinary isoflavone (daidzein, genistein, glycitein) and total isoflavone/CR increased significantly ($P < 0.001$) during soy intervention compared to baseline levels; this increase was observed in both Asians and non-Asians (Table 2 shows data for total isoflavones). Although baseline urinary isoflavone excretions did not differ between Asian and non-Asian participants (they were low for both groups), levels of the individual and total isoflavones were significantly higher among Asian compared to non-Asian participants during the intervention period (32.1 vs 12.8 μmol day$^{-1}$) ($P = 0.04$). The difference in urinary isoflavone levels between Asians and non-Asians diminished after adjustment for isoflavone intake (29.2 ± vs 17.1 μmol day$^{-1}$, $P = 0.16$). During the intervention period, the sources of isoflavones did not differ between the two groups. Soymilk, tofu and soybeans accounted for 46%, 50% and 4% respectively, of soy intake among Asian participants; the corresponding figures were 50%, 46% and 4% among non-Asian participants. Urinary isoflavone levels returned almost to baseline levels during the recovery months but they remained higher in Asian compared to non-Asian participants during the recovery period.

Cycle length did not change during the study period. The average cycle length was 29.2 ± 3.4 days during baseline, 29.3 ± 3.8 days during intervention and 29.5 ± 5.6 days during recovery. Both follicular and luteal phase length remained unchanged: the mean follicular phase length was 16.5, 16.7 and 17.3 days respectively, while the corresponding luteal phase length was 12.7, 12.5 and 12.2 days. The peak urinary LH level and the average follicular and luteal phase LH levels were not significantly different during intervention compared to baseline levels (data not shown). Results for cycle length and urinary LH were

 *© 2000 Cancer Research Campaign*

**Table 4** Summary of soy dietary intervention and serum hormone response in premenopausal women. Published studies are ordered by increasing dose of soy (mg of isoflavones per day) added during the intervention period

| Study | Blood specimen collection | Cycle length (intervention vs baseline) | Estradiol (intervention vs baseline) | Pg (intervention vs baseline) | SHBG (intervention vs baseline) | LH (intervention vs baseline) |
|---|---|---|---|---|---|---|
| Cassidy et al, 1994[a]<br>(1) 6 women in 1994<br>(2) 1 month<br>(3) soy protein<br>(4) 45 mg isoflavones day$^{-1}$<br>(5) 12.8 μmol day$^{-1}$ | Bloods every 3 days – early morning | T: ↑ 5.4%<br>F: ↑ 16.7%<br>L: ↓ 8% | F: ↑ 47.2%<br>L: no change<br>M: ↑ 9.9% | L: ↓ 14% | T: ↓ 2.6%<br>F: ↓ 1.8%<br>L: ↓ 5.2% | M: ↓ 66.5% |
| Petrakis et al, 1996<br>(1) 14 women<br>(2) 6 months<br>(3) soy protein isolate<br>(4) 74 mg isoflavones day$^{-1}$<br>(5) 69.8 μmol day$^{-1}$ | Variable | NA | T: ↑ 18% | T: ↓ 41% | T: ↓ 36% | NA |
| Duncan et al, 1999<br>(1) 14 women<br>(2) 3 cycles + 9 days<br>(3) soy protein powder, @ 3 doses<br>(4) control [C] = 10 mg isof day$^{-1}$<br>low soy [LS] = 64 mg isof day$^{-1}$<br>hi soy [HS] = 128 mg isof day$^{-1}$<br>(5) [C] = 4.9 μmol day$^{-1}$<br>[LS] = 23.4 μmol day$^{-1}$<br>[HS] = 49.8 μmol day$^{-1}$ | Bloods every other day beginning 7 days after LH surge in menstrual cycle 2 until end of diet period | LS vs C<br>T: ↑ 2.1%<br>MF: ↑ 3.6%<br>ML: no change<br><br>HS vs C<br>T: ↑ 1.4%<br>MF: ↓ 3.6%<br>ML: ↑ 5.5% | LS vs C<br>MF: ↑ 9.0%<br>ML: ↓ 6.4%<br><br>HS vs C<br>MF: ↑ 5.0%<br>ML: no change | LS vs C<br>MF: ↑ 22.4%<br>ML: ↓ 16.8%<br><br>HS vs C<br>MF: ↓ 3.9%<br>ML: ↓ 12.6% | LS vs C<br>No change<br><br>HS vs C<br>No change | LS vs C<br>MF: ↑ 1%<br>ML: ↓ 4.0%<br><br>HS vs C<br>MF: ↓ 5.6%<br>ML: ↑ 8.8% |
| Nagata et al, 1998<br>(1) 31 women on soy [S], 29 on control diet [C]<br>(2) 2 months<br>(3) soymilk<br>(4) 109 mg isofl day$^{-1}$ for [S]; 18 gm isofl day$^{-1}$ for [C]<br>(5) NA | Morning bloods on day 11 of cycles 1 and 3 | Entire group<br>[C]: ↓ 2.7%<br>[S]: ↑ 4.3%<br><br>Subgroup[b]<br>[C]: ↓ 1.7%<br>[S]: ↑ 5.1% | Entire group<br>[C]: ↑ 3.9%<br>[S]: ↓ 27.3%<br><br>Subgroup[b]<br>[C]:↑ 10.4%<br>[S]: ↓ 33.3% | NA | Entire group<br>[C]: ↑ 3.5%<br>[S]: no change<br><br>Subgroup[b]<br>[C]: ↑ 2.0%<br>[S]: ↓ 3.4% | NA |
| Lu et al, 1996<br>(1) 6 women<br>(2) 1 month<br>(3) soymilk<br>(4) 216 mg isofl day$^{-1}$<br>(5) NA | Blood samples before and 1 day after starting soymilk, at weekly intervals during soy feeding | T: ↑ 12.4% | T: ↓ 62%<br>LF: ↓ 81%<br>L: ↓ 49% | T: ↓ 35% | NA | NA |
| Wu et al (current study)<br>(1) 20 women<br>(2) 3 months<br>(3) tofu, soymilk, soybean<br>(4) 32 mg isofl day$^{-1}$<br>(5) 20.3 μmol day$^{-1}$ | Bloods on days 10–12 and 20–22, every cycle | T: no change<br>F: no change<br>L: no change | F: no change<br>L: ↓ 9.3% | F: no change<br>L: ↓ 13.9% | F: ↑ 1.5%<br>L: ↑ 2.0% | NA |

[a]These investigators reported in a letter (Setchell et al, 1995) that when results from three additional women were combined with those of the six subjects in Cassidy et al (1994), the increase in follicular phase plasma oestradiol levels in association with soy supplementation was smaller (↑ 16%) and was not statistically significant. [b]Subgroup included only women from whom blood samples were obtained on the same day or 1 day apart of the menstrual cycles 1 and 3. (1) Subjects[a]; (2) duration of soy feeding; (3) type of soyfood; (4) isoflavones (mg day$^{-1}$) during feeding; (5) urinary isoflavones (μmol day$^{-1}$) during feeding. T = total cycle; F = Follicular; l = Luteal; M = mid cycle; MF = midfollicular; ML = midluteal; LF = late follicular similar in Asians and non-Asians, and were comparable when we repeated the analysis restricted to the ‘clean’ dataset. Changes in serum oestradiol, progesterone and SHBG levels during the study are shown in Table 3. In all subjects combined, luteal phase oestradiol levels were reduced statistically significantly (–9.3%, $P = 0.05$) during soy intervention compared to baseline oestradiol level, but this was not observed for follicular phase oestradiol levels (–0.7%, $P = 0.92$). Luteal phase progesterone levels were also lower while SHBG levels were similar to baseline levels; none of these differences were statistically significant. Levels of oestradiol, progesterone and SHBG during recovery months did not differ significantly from baseline levels. Results were similar when we repeated the analyses restricted to the ‘clean’ dataset although the reduction in luteal phase oestradiol levels was reduced slightly (–8.9%, $P = 0.06$).

Although not in the original study design, we conducted ethnic-specific analysis because of the higher urinary excretion of isoflavones during intervention among Asians. Among Asian

© 2000 Cancer Research Campaign 

participants, both follicular (–7.4%) and luteal phase oestradiol (–17.4%) decreased in association with soy supplementation, although only the change during the luteal phase was statistically significant (Table 3). Among non-Asian participants, there were no reductions in serum oestradiol levels but there were statistically significant increases in both follicular and luteal phase SHBG levels (Table 3). Results were unchanged when we restricted the analysis to the 'clean' dataset. Urinary excretion of isoflavone was not, however, correlated with the difference in serum luteal oestradiol in all subjects combined ($P$ = 0.66) or separately in Asian ($P$ = 0.84) and non-Asian ($P$ = 0.95) participants.

### DISCUSSION

Intake of soy (in isoflavone units) in Asia was first reported to be around 150–200 mg per day (Cassidy et al, 1994). However, studies which provided details regarding the amounts of specific types of soy foods consumed and their corresponding isoflavone levels found a much lower intake in Japan and China, between 15 and 40 mg of isoflavones per day (Nagata et al, 1998; Chen et al, 1999; Wakai et al, 1999). The higher values initially reported appear to have been based on the assumption that soy foods contain 2–3 mg of isoflavones per gram, when these levels apply primarily to whole soybeans, a form of soy food not very commonly consumed in Asia (Adlercreutz et al, 1991; Nagata et al, 1998; Chen et al, 1999; Wakai et al, 1999). Recent studies (Franke et al, 1999; Murphy et al, 1999) confirmed that commonly consumed Asian soy foods (e.g. soymilk, tofu, miso) contain substantially lower isoflavone levels (0.10–0.35 mg $g^{-1}$) than whole soybeans; these values also tend to be lower than previously published values. Failure to normalize for molecular weight differences of the isoflavone isomers (Franke et al, 1999; Murphy et al, 1999) and inadequate accounting for moisture factor may explain in part higher isoflavone values. The average amount of isoflavones (32.0 mg $day^{-1}$) consumed by participants in our study during the intervention period is within the range of current isoflavone intake in Asia (Nagata et al, 1998; Chen et al, 1999; Wakai et al, 1999).

Soy may reduce the risk of breast cancer by affecting ovarian function, specifically endogenous oestrogen levels. This study provided some support that soy supplementation in the amount of about 30 mg of isoflavones per day over 3 months is associated with a statistically significant 9% reduction in luteal phase oestradiol. This reduction in serum oestradiol was confined to Asian participants who consumed more soy isoflavones (mean daily intake was 9 mg of isoflavones higher than non-Asians) and excreted higher isoflavone levels during intervention than non-Asian participants. However, it is not clear that this difference in reported soy intake contributed to the difference in hormone response, as there was no significant correlation between changes in serum oestradiol levels and urinary isoflavone levels in all subjects combined or separately in Asians and non-Asians. Only a larger study designed specifically to evaluate possible ethnic differences in hormonal response can resolve this question.

Table 4 summarizes the five published soy intervention studies in premenopausal women, showing differences in study design, including the type of soy products used, the amount of isoflavones added (range 45–216 mg) and the duration of supplementation. One was conducted among native Japanese and the other four were conducted presumably among non-Asians in the USA or the UK. Serum oestradiol was measured in all five studies, whereas progesterone, SHBG, LH and cycle length were measured in only some studies. The manner in which changes in ovarian function (i.e. timing and frequency of blood specimen collection) were monitored also varied.

Two studies, using soymilk in the amounts of 109 (Nagata et al, 1998) and 216 mg of isoflavones daily (Lu et al, 1996) provided strong support that soy supplementation may reduce serum oestradiol levels. However, given that isoflavone levels vary substantially in soymilk (even within a single brand) (Murphy et al, 1999) and it is unclear that the isoflavone content was monitored in these studies, we are less certain of the actual isoflavone intake. No significant reduction in serum oestradiol levels was found in all three studies which used soy protein or isolates (range 45–129 mg of isoflavones per day) (Table 4). One study measured both serum and urinary oestrogen levels; serum oestradiol levels did not change significantly (Duncan et al, 1999) but urinary excretion of oestrogens and the hypothesized genotoxic oestrogen metabolites (Xu et al, 1998) decreased significantly in association with soy supplementation. Reasons for the differences in blood and urinary oestrogen findings in this study are not apparent.

Interpretation of the current results is hampered by our lack of understanding of the relative importance of the source of and the amount of soy isoflavones and role of race/ethnicity in hormonal response. Isoflavone may not be the 'active' ingredient in soy foods or there may be other constituents (e.g. saponins, protease inhibitors) that are also important. While there are undisputed East–West differences in the consumption of soy foods and the epidemiologic evidence on cancer risk is based on soy foods (not soy ingredient), the majority of soy intervention studies have used soy ingredients because of greater ease in use and acceptance. One soy intervention study included both soy foods and soy ingredients as test foods (Cassidy et al, 1995); but because of the high salt content of miso (the soy food used), only three subjects completed this diet. Thus, there are no data from intervention studies that have used both soy foods and soy ingredients as test foods to assess whether the sources of isoflavones and/or differences in the amount of isoflavones or other factors are relevant. It will be important to design intervention studies that include both soy foods and soy ingredients in the same study and in which comparable amounts of isoflavones are added. In addition, it is important to measure isoflavone levels in the test soy foods to determine the actual amount of isoflavones consumed.

While it is reasonable to presume that the amount of isoflavones would matter, the optimal dose to use in intervention studies is not known. The almost fivefold range in the amount of isoflavones used in the published intervention studies may also contribute to the differences in study results.

Isoflavone bioavailability is known to depend on the relative degradation ability of gut microflora. The ingested isoflavones in soy foods are largely in the form of glycosides that are poorly absorbed in the small intestine compared with their aglycones because of their larger molecular weight and higher hydrophilicity (Brown, 1988). Glucosidases of intestinal microflora in the large intestine can hydrolyse the isoflavone glycosides to produce aglycones and promote their absorption. Deconjugating enzymes (i.e. β-glucuronidases and arylsulfatases) may also influence isoflavone bioavailability (Xu et al, 1995). Race/ethnicity may be associated with genetic variability in bacteria flora and/or differences in dietary habits (e.g. fat and fibre intake) which may influence the concentration of microfloral enzymes (Adlercreutz et al, 1992; Goldin and Gorbach, 1994; Xu et al, 1994; Tew et al, 1996;

© 2000 Cancer Research Campaign

Zhang et al, 1999) and consequently the metabolic fate of these compounds. For example, faecal bacterial β-glucuronidase activity has been found to increase in association with high dietary fat intake and to decrease in association with high dietary fibre intake (Goldin and Gorbach, 1994).

In this study, cycle length did not change in association with soy supplementation, whereas statistically significant increases of 5–12% have been reported (Cassidy et al, 1994; Lu et al, 1996; Nagata et al, 1998) (Table 4). Serum luteal or total progesterone levels decreased consistently in this and previous studies (13–41%) although this reduction did not reach statistical significance (Table 4). Serum SHBG decreased among Asian but increased statistically significantly among non-Asian participants in this study. There are no consistent changes in serum SHBG in previous studies (Table 4). Of previous studies that measured serum LH levels, a statistically significant reduction in levels was found in one (Cassidy et al, 1994). Urinary LH decreased nonsignificantly in association with soy intervention in this study (serum LH was not measured). Consumption of soy protein has been associated with significant reductions in total and LDL cholesterol and triglycerides, but the effect of soy on cholesterol levels may depend on the initial levels prior to intervention (Anderson et al, 1995). Serum lipid levels were normal to low in our participants; this may explain the absence of significant changes in lipid profile in this study.

Variation also exists in the monitoring of changes in hormone levels from one cycle to the next (Table 4). Unless daily or nearly daily blood specimens can be collected, it is difficult to be certain that comparisons of hormone levels between cycles are made on specimens collected from comparable days cycle to cycle. Our ability to identify day of LH peak as a marker of ovulation for over 90% of the menstrual cycles collected has enabled us to accurately determine day when specimens were collected relative to day of ovulation and to compare changes in each cycle with standardization for day of cycle.

In conclusion, supplementation using traditional soy foods may reduce serum oestradiol levels. Results from this and previous studies suggest that differences in the type of soy products (i.e. traditional soy foods versus soy protein products), amount of isoflavones, race/ethnicity of participants and variations in the monitoring of hormone changes have contributed to the divergent results. Larger studies of these questions are required.

### ACKNOWLEDGEMENTS

This work was supported by the California Breast Cancer Research Program (ITB-0091-L01), Special Institutional Grant (SIG 20) from the American Cancer Society, the Susan G Komen Breast Cancer Foundation and the Whittier Foundation. We thank all the study participants, Phuong Nguyen and Lydia Tran for their help with data collection and Dr Jean Hankin for her advice.

### REFERENCES

Adlercreutz H (1990) Western diet and western disease: some hormonal and biochemical mechanisms and associations. *Scand J Clin Lab Invest Suppl* **50**: 3–23

Adlercreutz H, Honjo A, Higashi A, Fotsis T, Hamalainen E, Hasegawa T and Okada H (1991) Urinary excretion of lignans and isoflavonoid phytoestrogens in Japanese men and women consuming a traditional Japanese diet. *Am J Clin Nutr* **54**: 1093–1100

Adlercreutz H, Mousavi Y and Hockerstedt K (1992) Diet and breast cancer. *Acta Oncol* **31**: 175–181

Anderson JW, Johnstone BM and Cook-Newell ME (1995) Meta-analysis of the effects of soy protein intake on serum lipids. *N Engl J Med* **333**: 276–282

Brown JP (1988) Hydrolysis of glycosides and esters. In: *Role of the Gut Flora in Toxicity and Cancer*, Rowland IR (ed), pp. 109–144. Academic Press: San Diego, CA

Cassidy A, Bingham S and Setchell KDR (1994) Biological effects of a diet of soy protein rich in isoflavones on the menstrual cycle of premenopausal women. *Am J Clin Nutr* **60**: 333–340

Cassidy A, Bingham S and Setchell (1995) Biological effects of isoflavones in young women: importance of the chemical composition of soyabean products. *Br J Nutr* **74**: 587–601

Chen Z, Zheng W, Custer LJ, Dai Q, Xhu XO, Jin F and Franke AA (1999) Usual dietary consumption of soy foods and its correlation with the excretion rate of isoflavonoids in overnight urine samples among Chinese women in Shanghai. *Nutr Cancer* **33**: 82–87

Coward L, Barnes NC, Setchell KDR and Barnes S (1993). Genistein, daidzein, and their b-glycoside conjugates: antitumor isoflavones in soybean foods from American and Asian diets. *J Agric Food Chem* **4**: 1961–1967

Duncan AM, Merz BE, Xu X, Nagel TC, Phipps WR and Kurzer MS (1999) Soy isoflavones exert modest hormonal effects in premenopausal women. *J Clin Endocrinol Metab* **84**: 192–197

Franke AA, Hankin JH, Yu MC, Maskarinec G, Low SH and Custer LJ (1999) Isoflavone levels in soy foods consumed by multiethnic populations in Singapore and Hawaii. *J Agric Food Chem* **47**: 977–986

Goldin BR and Gorbach SL (1994) Hormone studies and the diet and breast cancer connection. In: *Diet and Breast Cancer*, American Institute for Cancer Research, pp. 35–46. Plenum Press: New York

Israel R, Mishell DR Jr, Stone SC, Thorneycroft IH and Moyer DL (1972) Single luteal phase serum progesterone assay as an indicator of ovulation. *Am J Obstet Gynceol* **112**: 1043–1046

Krupps MA, Tierney LM Jr and Jawetz E (1982) *Physician's Handbook, 20th ed.* Lange Medical Publications: CA

Lu LJW, Anderson KE, Grady JJ and Nagamani M (1996) Effects of soya consumption for one month on steroid hormones in premenopausal women: implications for breast cancer risk reduction. *Cancer Epidemiol Biomarkers Prev* **5**: 63–70

Messina M and Barnes S (1991) The role of soy products in reducing risk of cancer. *J Natl Cancer Inst* **83**: 541–546

Murphy PA, Song TT, Buseman G and Barua K (1997) Isoflavones in 8 soy-based infant formulas. *J Agric Food Chem* **45**: 4635–4638

Murphy PA, Song TT, Buseman G, Barua K, Beecher GR, Trainer D and Holden J (1999) Isoflavones in retail and institutional soy foods. *J Agric Food Chem* **47**: 2697–2704

Nagata C, Kabuto M, Kurisu Y and Shimizu H (1997) Decreased serum estradiol concentration associated with high dietary intake of soy products in premenopausal Japanese women. *Nutr Cancer* **29**: 228–233

Nagata C, Takatsuka N, Inaba S, Norito K and Shimizu H (1998) Effect of soymilk consumption on serum estrogen concentrations in premenopausal Japanese women. *J Natl Cancer Inst* **90**: 1980–1985

Petrakis N, Barnes S, King EB, Lowenstein J, Wiencke J, Lee MM, Miike R, Kirk M and Coward L (1996) Stimulatory influence of soy protein isolate on breast secretion in pre- and postmenopausal women. *Cancer Epidemiol Biomarkers Prev* **5**: 785–794

Scott JZ, Stanczyk FZ, Goebelsmann U and Mishell Dr Jr. (1978) A double-antibody radioimmunoassay for serum progesterone using progesterone-3-(O-carboxymethyl) oximino[$^{125}$I]-iodohistamine as radioligand. *Steroids* **31**: 393–405

Setchell KDR, Cassidy A and Bingham S (1995) Reply to AH Wu and MC Pike (letter). *Am J Clin Nutr* **62**: 152–153

Stanczyk FZ, Shoupe D, Nunez V, Macias-Gonzales P, Vijod MA and Lobo RA (1988) A randomized comparison of normal estradiol delivery in post-menopausal women. *Am J Obstet Gynecol* **159**: 1540–1546

Tew BY, Xu X, Wang HJ, Murphy PA and Hendrich S (1996) A diet high in wheat fiber decreases the bioavailability of soybean isoflavones in a single meal fed to women. *J Nutr* **126**: 871–877

Wakai K, Egami I, Kato K, Kawamura T, Tamakoshi A, Lin Y, Nakayama T, Wada M and Ohno Y (1999) Dietary intake and sources of isoflavones among Japanese. *Nutr Cancer* **33**: 139–145

Wang HJ and Murphy PA (1994) Isoflavone content in commercial soybean foods. *J Agric Food Chem* **42**: 1666–1673

Wu AH and Pike MC (1995) Dietary soy protein and hormonal status in females. (letter). *Am J Clin Nutr* **62**: 151–152

© 2000 Cancer Research Campaign

Xu X, Wang HJ, Murphy PA, Cook L and Hendrich S (1994) Daidzein is a more bioavailable soymilk isoflavone than is genistein in adult women. *J Nutr* **124**: 825–832

Xu X, Harris KS, Wang HJ, Murphy PA and Hendrich S (1995) Bioavailability of soybean isoflavones depends upon gut microflora in women. *J Nutr* **125**: 2307–2315

Xu X, Duncan AM, Merz BE and Kurzer MS (1998) Effects of soy isoflavones on estrogen and phytoestrogen metabolism in premenopausal women. *Cancer Epidemiol Biomarkers Prev* **7**: 1101–1108

Zhang Y, Wang GJ, Song TT, Murphy PA and Hendrich S (1999) Glycitein is a more bioavailable isoflavone than is daidzein in humans having moderate fecal isoflavone degradation activity. *J Nutr* **129**: 967–962

*© 2000 Cancer Research Campaign*
